# Managing Terrorism or Accidental Nuclear Errors, Preparing for Iodine-131 Emergencies: A Comprehensive Review

**DOI:** 10.3390/ijerph110404158

**Published:** 2014-04-15

**Authors:** Eric R. Braverman, Kenneth Blum, Bernard Loeffke, Robert Baker, Florian Kreuk, Samantha Peiling Yang, James R. Hurley

**Affiliations:** 1 Department of Psychiatry, College of Medicine, University of Florida and McKnight Brain Institute, Gainesville, FL 32610, USA; E-Mail: ericb1957@gmail.com; 2 Department of Clinical Neurology, PATH Foundation NY, New York, NY 10010, USA; E-Mails: helpingotherstoday@comcast.net (B.L.); fkreuk.path@gmail.com (F.K.); 3 Department of Neurosurgery, Weill-Cornell Medical College, New York, NY 10065, USA; 4 Department of Biological Sciences, Texas Tech University, Lubbock, TX 79409, USA; E-Mail: Robert.Baker@ttu.edu; 5 Natural Science Research Laboratory, Museum of Texas Tech University, Lubbock, TX 79409, USA; 6 Department of Endocrinology, National University Hospital of Singapore, Singapore 119228; E-Mail: Peiling_Yang@nuhs.edu.sg; 7 Department of Medicine, Weill Cornell Medical College, New York, NY 10065, USA; E-Mail: jrh2004@med.cornell.edu

**Keywords:** iodine-131 (^131^I), potassium iodide (KI), nuclear terrorism, accidental errors, uranium fission, children, plume radius

## Abstract

Chernobyl demonstrated that iodine-131 (^131^I) released in a nuclear accident can cause malignant thyroid nodules to develop in children within a 300 mile radius of the incident. Timely potassium iodide (KI) administration can prevent the development of thyroid cancer and the American Thyroid Association (ATA) and a number of United States governmental agencies recommend KI prophylaxis. Current pre-distribution of KI by the United States government and other governments with nuclear reactors is probably ineffective. Thus we undertook a thorough scientific review, regarding emergency response to ^131^I exposures. We propose: (1) pre-distribution of KI to at risk populations; (2) prompt administration, within 2 hours of the incident; (3) utilization of a lowest effective KI dose; (4) distribution extension to at least 300 miles from the epicenter of a potential nuclear incident; (5) education of the public about dietary iodide sources; (6) continued *post-hoc* analysis of the long-term impact of nuclear accidents; and (7) support for global iodine sufficiency programs. Approximately two billion people are at risk for iodine deficiency disorder (IDD), the world’s leading cause of preventable brain damage. Iodide deficient individuals are at greater risk of developing thyroid cancer after ^131^I exposure. There are virtually no studies of KI prophylaxis in infants, children and adolescents, our target population. Because of their sensitivity to these side effects, we have suggested that we should extrapolate from the lowest effective adult dose, 15–30 mg or 1–2 mg per 10 pounds for children. We encourage global health agencies (private and governmental) to consider these critical recommendations.

## 1. Introduction

The fission of uranium produces large amounts of iodine-131 (^131^I) that may be released into the atmosphere in the course of a nuclear accident (2.878% yield of uranium-235) [1]; the resultant plume of radioactivity can travel as far as 300 miles. Iodine-131 can enter the body by inhalation or by ingestion of contaminated food or milk. The nuclear-reactor incidents at Chernobyl (1986), Three Mile Island (1979) and Fukushima (2011) provide a sense of urgency and concern over the possible hazards of ionizing radiation to the thyroid from ^131^I. This isotope, with a half-life of 8 days, is one of the most dangerous ones released in a nuclear accident, because it is concentrated in the thyroid gland and emits beta rays which cause cellular damage. The development of benign and malignant thyroid nodules following exposure to ionizing radiation from the atomic explosions at Hiroshima and Nagasaki has been documented by Hollingsworth [2] and others [3,4].

Potassium iodide prophylaxis can significantly reduce the radiation dose to the thyroid due to exposure to ^131^I. In an experimental situation potassium iodide (KI) given prior to exposure reduced the accumulation of ^131^I in the thyroid gland from an average of 20% to less than 2% [5]. At the time of the Chernobyl reactor accident, the Polish government distributed KI to 95% of Polish children and 23% of the total population. It was estimated that the projected thyroid dose of individuals who were given KI was reduced by nearly 40% [6]. However it is critical that KI be taken within a few hours of exposure. After 3-4 hours the effectiveness is decreased by 50%.

## 2. Review and Recommendations

After 9/11, the Public Health Security and Bioterrorism Preparedness Act of 2002 was passed, which through Section 127 required states to consider using potassium iodide (KI) in radiological emergency response plans, within 20 miles of a Nuclear Power Plant (NPP) [7]. Section 127 was suspended in 2008 by the Office of Science and Technology Policy through the invoking of a waiver by Science Advisor John Marburger III [8]. Since suspension of Section 127 and with impetus from the 2011 Fukushima calamity, we undertook a comprehensive review of the scientific literature, recommendations and guideline from various health and governmental agencies regarding the risks to the community of exposure to ionizing radiation and the use of potassium iodide prophylaxis. Recommendations from the following organizations were reviewed; they included the American Thyroid Association (ATA), the Food & Drug Administration (FDA), the Centers for Disease Control and Prevention (CDC), the United States Nuclear Regulatory Commission (USNRC), the United Nations (UN), the National Council on Radiation Protection & Measurements (NCRP) and the World Nuclear Association. Based on this review we are proposing that at minimum, the ATA recommendation of 50-mile pre-distribution of KI and availability be implemented and the above agencies issue new protective guidelines. 

### KI Agency Recommendations

The ATA has advocated that KI tablets be part of an emergency plan in response to a radiological event, available up to 200 miles of a NPP, pre-distributed within 50 miles of a NPP, and used only under regulatory guidance [9,10]. Poland utilized KI tablets in response to the Chernobyl accident [11]. This historical instance has served as a strong practical demonstration of the effectiveness of KI usage post radiological event [9]. The ATA endorsed the FDA’s 2001 KI dosage and usage recommendations. One 130 mg tablet is recommended for adults over 40 years with a very high predicted thyroid gland exposure of ≥500 cGy. For other adults, 18 to 40 years with a predicted thyroid gland exposure of ≥10 cGy and pregnant or lactating women with a predicted thyroid gland exposure of ≥5 cGy, a dose of 130 mg KI is also recommended. Adolescents and children, 3 to 18 years, with a predicted thyroid gland exposure of ≥5 cGy, a dose of 65 mg KI is recommended; if fully grown, adolescents are recommended the adult dose of 130 mg KI. For children, 1 month to 3 years with a predicted thyroid gland exposure ≥5 cGy, a dose of 32 mg KI is recommended and for infants, birth to 1 month, with a predicted thyroid gland exposure of ≥5 cGy a 16mg KI dose is recommended [12]. The impracticality of these recommendations is recognized by the authors, for by the time the necessary data has been collected and dosimetric calculations have been made and communicated, it would be too late for effective prophylaxis. Thresholds of exposure are very important determining factors in the decision to include KI in the radiological emergency response.

The WHO recommends KI be taken under regulatory guidance and stresses the importance of the thyroid blocking in children and pregnant/nursing women [13,14]. There are two main differences in the KI recommendations of the WHO as compared to the FDA; specifically, adolescents over 12 years of age are recommended the 130 mg KI dose by the WHO, and the WHO’s recommended threshold of exposure before prophylaxis for pregnant and lactating women and children 18 and younger is 1 cGy [15]. The WHO endorsed public health officials and authorities to provide proper guidance on when to take KI and the dosage regimen for KI usage [13]. Furthermore, the WHO states that the effectiveness of thyroid blocking depends on the timely administration of KI tablets. When taken three to four hours after exposure the effectiveness of KI thyroid blocking is reduced by 50% [13].

## 3. Potassium Iodide Prophylaxis Review

### 3.1. Potassium Iodide Prophylactic Historical Success

KI has been utilized as a prophylaxis in radiological emergency preparedness plans in the United States, in part, because of its reported historical success. The Polish government ordered the creation and usage of KI by its national pharmacies three days following the Chernobyl nuclear accident [6]. Within 24 h of this decision, in certain areas, 75% of Polish children had received KI [15]. Ultimately, KI was distributed to 95% of Polish children and 23% of the total population [6]. It was estimated that due to the KI prophylaxis of the general public, Poland was able to reduce the projected thyroid dose of individuals that were given KI, within 24 h of the Polish government’s decision to utilize KI, by nearly 40% [6,11]. Poland’s success in radioiodine blocking stemmed from the considerable amount of time it had to respond to the Chernobyl accident. If the nuclear accident had occurred within Poland, the radioiodine blocking effect would be essentially 0%. While this serves as a great example of preventative success of KI in radioiodine blocking, it also stresses the importance of KI pre-distribution to areas surrounding nuclear power plants that can experience significant radiological exposures within an extremely short period of time.

### 3.2. Potassium Iodide Prophylaxis—Lowest Effective Prophylactic Dose

Prior to the 1986 Chernobyl reactor accident, the United States of America (USA) Food & Drug Administration’s (FDA) recommendation for ^131^I prophylaxis was 130 mg per day of stable iodide (SI) for adults and children above 1 year of age and 65 mg per day for children below 1 year of age [15]. However, research by Sternthal *et al*., in 1980 [5] evaluated thyroid radioiodine uptake in euthyroid volunteers using a systematic dose—response regimen of a single dose daily for twelve days of between 10 and 100 mg of sodium iodide. Sternthal *et al*. found that single doses at 30, 50 and 100 mg suppressed 24-hour thyroid uptake of ^131^I to 0.7 to 1.5 percent. Continued administration of 15 mg up to 100 mg of iodide daily resulted in 24 h uptake values below 2 percent [5]. Moreover, Sternthal *et al*. determined that continued administration of KI (8 and 12 days) in prophylaxis led to significant changes in thyroid hormones, inducing significant reductions in triiodothyronine (T3) and serum thyroxine, as well as increases in serum thyroxine (TSH) concentrations [5]. The values for serum TSH in some of the subjects receiving 100 mg of sodium iodide, daily, were slightly above the normal range on days eight and twelve [5]. However, after the withdrawal of iodide, all values returned to those observed during the control period [5]. Interestingly, Thompson *et al*. showed that a KI dose as little as 6 mg is adequate to reduce basal metabolic rate of subjects; however, no thyroid uptake was measured in this study [16].

The FDA states that the effectiveness of KI in thyroidal radioiodine blockage is well established; however, based upon a review of the efficacy of KI in prophylaxis, a recommendation of 130 mg KI may be excessive, especially in adolescents and children [17]. Furthermore, lower dosages have been shown to be effective and adequate. In an early case study, there was a significant reduction in ^131^I thyroidal dose over a 14 day period in euthyroid adolescents and young adults given very low stable iodide dosages [17,18]. For example, two adolescents aged 8 and 9, ~50 lbs, were administered 1.8 mg stable iodide daily and administered an oral dose of ^131^I for 14 days, which corresponded with a 33% and 48% reduction in thyroid dose, respectively [17,18]. Undergoing the same radioiodine treatment, two young adults, aged 22 and 23, administered only 4.2 mg stable iodide daily were able to reduce the thyroid dose by 69% and 62%, respectively [17,18]. While the dosages at this level are inadequate to effectively block radioiodine uptake, they do provide information regarding the lower limits of an effective dose. Subsequent studies have determined that 20 mg of KI has been shown to be over 90% effective in blocking radioiodine thyroidal uptake in certain individuals [17]. Furthermore, 30 mg of KI taken at the time of radioiodine exposure and a full week of 15 mg dosing is up to 98%–99% effective in averting thyroid dose in 70 kg euthyroid individuals [5]. These studies implicate 20–30 mg SI dosages for certain individuals as being adequate and effective [5,17]. There are yet to be sufficient studies in infants, children and adolescents, and so we have postulated that a weighted dosage, 1 mg KI per 10 lbs for iodine sufficient individuals up to 2 mg KI per 10 lbs for iodine insufficient individuals, will be effective in blocking radioiodine uptake. 

It has been suggested that a larger KI dose is more effective for periods of extended exposure risk. Individuals with thyroids of lesser volume and higher activity and with lower plasma iodide concentrations will experience increased thyroid clearance and subsequently will be more vulnerable to radioiodine exposure [17]. Increased clearance necessitates larger dosages of KI or more frequent dosing at lower KI dosages. Interestingly, averted dose was not improved with dosages exceeding 100 to 200 mg KI [17]. The necessity for continued prophylaxis depends on the risk for continued exposure. Thyroidal iodine turnover rate is 1% in adults, 17% in iodine-replete neonates, and as high as 62% and 125% in cases of moderate and severe iodine deficiency [19]. An accelerated iodine turnover rate would necessitate continued prophylaxis for children and iodine deficient persons in instances in which risk for continued exposure persists. The neonate is hypersensitive to iodine loading due to its small pool of intra-thyroidal iodine because the Wolff-Chaikoff effect occurs at a critical ratio between intrathyroidal iodine and total iodine [17]. Prolonged prophylaxis in neonates requires additional investigation.

While a 100 mg KI dose provides thyroidal blocking benefits exceeding 24 h, a similar effect can be seen by continued prophylaxis at lower dosages [17]. For example, a 100 mg KI dose taken 48 h before exposure corresponds with a 78% averted dose; taken 72 h before exposure, 100 mg KI corresponds with a 25% averted dose [17]. Notably, 25 mg of KI taken 48 h before exposure did not block thyroidal radioiodine uptake [17]. Taken just prior to radioiodine exposure by a euthyroid adult, 100 mg of KI is at least 95% effective in blocking radioiodine uptake [17]. If risk of exposure persists over 24h, 15 mg of KI taken subsequent days will reduce radioiodine uptake by 90% [17]. This example indicates that while a large initial dose does provide opportunities for continued prophylaxis, maintenance of lower dosages of KI are more effective in long-term thyroid dose aversion. Interestingly, a small study (*n* = 9) evaluated the long-term efficacy of a lowered dose, which determined that when administered 24 h before radioiodine exposure, 37 mg stable iodide was able to reduce the thyroidal dose by 86% [17,20]. This example shows the unique result, in which a low dosage was able to substantially block uptake 48 h after ingestion. Differences in iodine sufficiency explain this data.

In order to facilitate initial prophylaxis to affected populations within the first two hours of a ^131^I exposure, it may be prudent to reduce the costs of emergency preparedness. Current ATA recommendations for KI intake after radiation are based on the use of 130 mg tablets. While we are not discounting the efficacy of 100 mg or more as a suppressant of ^131^I uptake by the thyroid, it seems logical to investigate and initiate lower doses especially for children. This will not only reduce the already limited side effects of KI prophylaxis, but may importantly, reduce costs for this emergency preparedness initiative without sacrificing KI prophylactic effectiveness. Finally, the possibility of other more cost-effective preparations should be considered. Potassium iodide granules should be considered for prophylactic use instead of the currently recommended potassium iodide tablets, because they have an extended shelf life of 20 years, compared to, the 5–7 year shelf-lives of potassium iodide tablets. It is likely that the shelf life of KI tablets is indefinite if properly stored.

According to Sternthal *et al*., [5] it is extremely important that, in the event of a radiological incident the iodide be administered promptly, to prevent as effectively as possible radioiodine accumulation in the thyroid gland. The time limit for the effectiveness of KI prophylaxis is summarized in Table 1.

**Table 1:** The percentage efficacy of 100mg Potassium Iodide prophylaxis in relation to the time of administration and radioiodine exposure.

Time of KI prophylaxis	Effectiveness
96 h before radioiodine exposure	5%
72 h before radioiodine exposure	32%
48 h before radioiodine exposure	75%
24 h before radioiodine exposure	93%
At radioiodine exposure	99%
1–2 h after radioiodine exposure	85%–90%
3–4 h after radioiodine exposure	50%
8 h after radioiodine exposure	40%
24 h after radioiodine exposure	7%

*Source*: modified from Sternthal *et al*.; World Health Organization; Becker and Zanzonico; Zanzonico and Becker [5,6,13,21,22].

A judicious and cost-effective method to determine the quantity of tablets to be distributed may be to base on the aggregate family weight, and an eight day dosage regimen. The weight of the family is the primary analytic, used to determine the number of tablets to be distributed. An eight day dose response regimen is necessary for an instance in which evacuation is impossible, and sheltering is necessary. Based on the Sternthal *et al*. [5] study we recommend the following dosage regimen as useful prophylaxis that could suppress ^131^I uptake by the thyroid. An initial dose of 2 mg of stable iodide per 10 pounds taken, on the first day of radioiodine exposure and a protracted daily dose of 1 mg stable iodide per 10 pounds, taken based on the risk for continued exposure. 

Considering that a mg/pound recommendation may be too complicated for routine use, a simplified plan has been determined, which utilizes the principles of a lowest effective prophylaxis; dosages dependent on a 15 mg KI tablet. The plan has been summarized in Table 2, in which 45 mg is utilized for individuals weighing greater than 200 lbs, 30 mg of KI is utilized in the weight range 100 to 200 lbs, 15 mg KI is utilized for individuals weighing 50 to 100 lbs, and 7.5 mg KI is utilized for individuals weighing less than 50 lbs. A scored 15 mg KI tablet is user friendly and can improve dosing regimens for infants, children and adolescents, making it simpler to provide effective dosages for these age groups. These dosages are taken on the first day; continued use depends on risk for repeat exposure following the Sternthal *et al.* [5] finding that after the initial dose 15 mg daily gave continued adequate suppression. 

**Table 2:** Author recommendations for Potassium Iodide prophylactic dosage and number of tablets for different weight classes and age groups.

Age Group	Individual Weight	KI Dosage	Number of KI 15 mg Tablets
Adults over 18 years	> 200 lbs	45 mg	3
Between 12–18 years	> 200 lbs	45 mg	3
Adults over 18 years	100–200 lbs	30 mg	2
Between 12–18 years	100–200 lbs	30 mg	2
Between 3–12 years	100–200 lbs	30 mg	2
Between 12–18 years	50–100 lbs	15 mg	1
Between 3–12 years	50–100 lbs	15 mg	1
Between 3–12 years	< 50 lbs	7.5 mg	½
Birth to 3 years	< 50 lbs	7.5 mg	½
Pregnant or lactating	-	45 mg	3

* Based on 1–2 mg per 10 lb weighted dosages. For euthyroid 150 lb adults 10 mg of stable iodine taken at radioiodine exposure is 87%–88% effective in blocking thyroidal radioiodine uptake and 30 mg of stable iodine taken on the first day of exposure, with 15 mg taken each subsequent day of exposure is 98%–99% effective in blocking radioiodine uptake [5].

Educational retention of proper KI usage can be improved if individuals pick up KI tablets themselves from distribution centers where they are able to speak with educated health professionals and receive proper educational materials [23]. Education retention is an important factor in assessing the appropriate distribution methods for KI.

The 130 mg KI dose (of two 65 mg pills) is a consensus dose of thyroid specialists adopted by various agencies that has never been studied in infants, children and adolescents, our target population. The lowest effective dose in the medical literature can vary from as little as 4 to 20 mg of KI (for a ~70 kg person) [16,17,18]. It is prudent to choose a 15 mg dose, because it is already available and easily produced, distributed, and administered. 

A 15 mg scored pill, and a seven-day prophylaxis of 1–2 mg of KI per 10 pounds, is ideal. It will prevent panicking parents from cutting 65 mg tablets and dosing in error, causing hypothyroidism or other side effects. It will provide sustained protection for one full week against 131 I and has not been associated with any problems [5]. Furthermore, most parents will not be confident in taking two 65 mg pills on the first day of a nuclear accident. Daily 15 mg dosing that is adjusted according to the child’s weight is simple, user-friendly, and believable. Moreover, a 15 mg tablet is adaptable even for infants; it can be broken and crushed easily. Since our infants, children and adolescents are most vulnerable to radioiodine exposure and excessive iodine intake, it is prudent that a lowered dose be used.

The instructions of current KI prophylactic labels are unclear and may lead to over-dosing. 65 mg KI tablet instructions state that the pill will work for ~24 h. With risk for continued exposure, individuals will keep taking these high dosage KI tablets. A week of daily prophylaxis at 130 mg KI or 910 mg KI weekly will increase the side effects from prophylaxis while not significantly increasing efficacy in thyroid blockage [5]. The range in the efficacy of KI prophylaxis is dependent on the variable iodine status of individuals and other factors such as thyroidal turnover rate [19]. Therefore, we recommend 1 mg KI per 10 lbs for iodine sufficient individuals up to 2 mg KI per 10 lbs for iodine insufficient individuals. 

The 1–2 mg KI per 10 lb recommendation is a cautionary dosage to limit side effects from excess dosing, albeit the efficacy in thyroidal radioiodine blockage may be improved with a higher initial dose [5,17]. While our recommendation reduces thyroid uptake by 90%, improvement in the target thyroid uptake suppression from 95%–98% necessitates dosages of at least 30 mg iodide for euthyroid 150 lb adults [5]. In regards to continued daily dosing following an initial dose, there is improvement in efficacy with a larger dose. For example, after an initial dose of 30 mg iodide continued daily dosing at 15 mg iodide continued to reduce thyroid uptake to 0.8%–1.5% at 24 h and 1.9% at 12 days; however, continued daily dosing at 10 mg iodide reduced thyroid uptake by 4% at 12 days [5]. Lower dosages of KI can be effective in individuals who are iodine sufficient. 

### 3.3. Iodine-131 Biological and Physical Analysis

Radioactive atoms are unstable due to an excess of energy in their nucleus. In order to reach a more stable state some of the excess energy is given off (emitted) in process called radioactive decay. After undergoing radioactive decay most atoms (including ^131^I) are no longer radioactive. Every radioactive atom has a specific time interval during which half of the atoms decay called a physical half-life. For ^131^I the physical half-life is 8 days. When ^131^I decays a neutron is converted to a proton and an electron. The electron is ejected from the nucleus as a beta particle and the rest of the excess energy is emitted as gamma rays. Beta particles have both mass and a negative electrical charge. They interact with surrounding tissue and transfer all of their energy within 1–2 mm. Beta rays are responsible for the thyroid damage caused by ^131^I. Gamma rays are electromagnetic radiation and have no physical mass or electrical charge. They penetrate tissue easily and can be detected outside of the body. Instruments that can detect and quantify gamma radiation can be used to determine the amount of ^131^I in the thyroid gland [24].

Iodine is readily absorbed from the intestinal tract and the lungs. It is transported in the bloodstream to the thyroid where it is concentrated and used to make the thyroid hormones T4 and T3. These are stored in the lumen of thyroid follicles for a prolonged period before being secreted into the bloodstream. Only about 1% of the stored iodine is secreted daily [21,25]. Iodine-131 is chemically identical to stable iodine and follows a similar pathway. Because of its relatively short physical half-life (8 days) and prolonged retention it delivers almost all of its radiation to the thyroid and the radiation dose is proportional to the uptake [21]. If enough is concentrated in the thyroid the radiation absorbed by the thyroid cells will be high enough to kill them [26]. This can be beneficial in patients with overactive thyroid glands and thyroid cancer, and ^131^I is used extensively to treat such patients. At lower radiation doses the cells may remain viable and radiation induced mutations may result in malignant transformation [27].

KI prophylaxis floods the body with stable iodine that competes with ^131^I for transport into the thyroid. Normally there is very little stable iodine in the body. If a small amount of radioiodine is given to a normal person, about 30% will be concentrated in the thyroid. After KI prophylaxis the radioiodine is diluted with stable iodine and the thyroid uptake will be almost completely blocked. The ingested or inhaled ^131^I that is not concentrated in the thyroid is rapidly excreted in the urine with a smaller amount excreted in the feces, perspiration, and, in a lactating female in the milk [17,21]. It must be stressed that to be effective KI prophylaxis must be started within one hour of exposure to ^131^I.

### 3.4. Shelf-Life of Potassium Iodide

The shelf-life of KI refers to the period in which a drug manufacturer can guarantee the full potency of KI. The shelf-life of the predominantly used KI tablets IOSAT^TM^ 130 mg and Thyrosafe ^TM^ 65 mg is seven and six years, respectively [12]. The liquid formulation of KI has a shelf life of five years [12]. There is very limited information regarding the establishment of these various shelf-lives, and this salt, being chemically stable, probably has an indefinite shelf-life if correctly stored. Capsulation will lead to an indefinite shelf-life.

Furthermore, a drug can still be good and safe to use past an expiration date. A 2006 study conducted by Lyon *et al.*, investigated the stability profiles of drug products extended beyond labeled expiration dates and found that KI tablets and granules remained active, for longer than their expiration date [28]. KI tablets remained active 28–184 months longer than the set expiration date, with an average extension time of 69 months, and KI granules remained active 225–278 months longer than the set expiration date, with an average extended time of 254 months [28]. KI granules showed the greatest extension of the expiration date of nearly 20 years in all cases and, therefore, should be considered as a potential prophylactic source [28]. Capsulation of KI granules can be the vehicle for implementation.

In a study determining the relative abundance of iodine in iodized salt, iodine content was studied in relation to various environmental conditions. Since, iodide is oxidized to iodine that due to high volatility can readily sublime as temperature increases; both heat and moisture have been implicated as causing loss of iodine content in iodized salt [29]. If salt is stored in a location with relative humidity greater than 65%, its iodine content will follow an exponential loss pattern [29]. Tests involving 95% relative humidity reduced the iodine content of salt samples to essentially 0 mg I/kg in as little as 25 days [30]. Biber *et al.* reported a 58.5% iodine loss at 30%–45% relative humidity over 3.5 years in sealed paper bags [30]. The ideal relative humidity level in a home is between 40%–50%, so this iodine loss will take place, thus, iodized salt requires cool, watertight storage. 

Since the iodine in salt is actually KI, this study signifies the importance of regulating temperature in KI storage, limiting moisture contact, and storing KI in limited light to extend the shelf-life of the drug [28,31]. It is recommended that KI be dispensed in opaque sealed containers containing a desiccant, thereby effectively mitigating sources of iodine loss. Furthermore, KI granules, with a theoretical 20 year extended shelf-life compared to the 5–7 year shelf-life of presently used KI tablets, may be a better source for prophylaxis [28]. It is widely accepted that salt crystals do not expire. Furthermore, utilizing KI granules, with the extended shelf life, can mitigate the reduction in shelf life that occurs in distributing a lower KI dosage. The increased shelf life would reduce the costs associated with restocking KI distribution centers and stockpiles, and it would increase the likelihood that families will maintain a KI stock within its expiration date. Liquid formulations of KI should be considered for use in toddlers and neonates. 15mg scored pills allow for easiest compliance and flexibility. 

### 3.5. Potential Use of Food for Iodine Prophylaxis

The ATA and other informational agencies should emphasize the potential for alimentary sources of iodine to be used in the absence of KI tablets. Seaweed, especially kelp is the best natural source of iodine [32,33]. Table 3 lists the iodine concentration in various common foodstuffs. Seaweed (preferably kelp) is the only possible dietary substitute for KI in prophylaxis. The pharmacokinetics of kelp and bioavailability of iodine after oral ingestion has not been readily studied; this information would offer valuable assessments of kelp as an alternative to potassium iodide in prophylaxis. Interestingly, ^131^I uptake is reduced when the supply of alimentary iodine is increased [34]. This effect has been observed and confirmed in several studies [21,35,36]. Importantly, there is high variability in the iodine content of alimentary sources, dependent on where and when the foodstuff is produced. Teas J. *et al.* conducted an analysis of the iodine content variability in commercial seaweeds and determined that the iodine content of 12 different seaweed samples ranged from 16 mcg I/gram seaweed ± 2 mcg I/g (in nori seaweed) to 8,165 mcg I/g seaweed ± 373 mcg I/g (in *Laminaria digitata* kelp granules) [37]. With the exception of seaweed, especially kelp, the foods listed in Table 3 should not be used for prophylaxis; rather, these foods are valuable for maintaining iodine sufficiency. 

When undergoing prophylaxis, the human body experiences excessive iodine uptake. The excess iodine works competitively with radioiodine to limit thyroidal radioiodine uptake.

**Table 3:** Iodine concentrations in foodstuffs.

Food	Iodine Concentration	Food	Iodine Concentration
Kelp Flakes *	13 mg/tsp	Iodized Table Salt	0.3 mg/tsp
Kombu Seaweed *	4 mg/tsp	Fish Sticks	0.2 mg/6 fish sticks
Laminaria Kelp *	3 mg/tsp	Shrimp	0.2 mg/lb
Hijiki *	0.5 mg/gram	Baked Turkey Breast	0.2 mg/lb
Himalayan Crystal Salt	2 mg/tsp	Plain Yoghurt	0.15 mg/lb
Life-flo Liquid Plus	1 mg/20 drops	Canned Tuna	0.1 mg/lb
Cod or Haddock	0.5 mg/lb	Navy Beans	0.1 mg/cup
Lobster	0.45 mg/lb	Baked Potato	0.1 mg/med. Potato
Cranberries	0.4 mg/4oz		

Note: * indicates the source could be useful in alimentary radioiodine prophylaxis.

### 3.6. Iodine Deficiency and Dietary Iodine

Thyroidal uptake of radioactive iodine is higher in people with iodine deficiency than in people with iodine sufficiency. For this reason, iodine-deficient individuals have a higher risk of developing radiation-induced thyroid cancer when exposed to radioactive iodine [21]. Studies in the Bryansk region of Russia after the Chernobyl accident showed that the excess relative risk of thyroid cancer related to ^131^I exposure was twice as high in areas with severe iodine deficiency as in areas with normal iodine intake [38]. An inverse relationship was discovered between levels of urinary iodine excretion and the Excess Relative Risk (ERR) of thyroid cancer in these persons, indicating that iodine sufficiency indeed helps mitigate thyroid cancer risk [38]. Chronic iodine deficiency itself may be associated with an increased risk of goiter and the follicular form of thyroid cancer [39,40,41]. By ensuring adequate intake we can prepare for a possible nuclear event as well as improve public health. 

In 2006 The Institute of Medicine set the Recommended Dietary Allowance (RDA) for iodine at 150 μg per day in adult men and women, 100 μg per day for infants between 0 and 6 months, 130 μg per day for children between 6 and 12 months, 90 μg per day for children between 1 and 5 years old, 120 μg per day for children between 5 and 12 years old, 150 μg per day for adolescents over age 12, 220 μg per day for pregnant women and 290 μg per day for lactating women [42]. Because the effects of iodine deficiency are most severe in pregnant women and their babies, the American Thyroid Association has recommended that women who are in the preconception period, pregnant or breastfeeding in the U.S. and Canada take a prenatal multivitamin containing 150 μg iodine per day [43]. Individuals who add table salt to their food regularly should use iodized salt. One teaspoon of iodized salt contains approximately 400 μg iodine. Most iodine-containing multivitamins have at least 150 μg iodine, but only about half of the types of multivitamins in the U.S. contain iodine. [44]. In air, iodide is oxidized and can readily sublime with heat, so KI tablets and iodized table salt should be stored in airtight containers free from humidity and heat to reduce iodine losses [29]. Table 4 lists good dietary sources of iodine. It is assumed that the fast food and prepared food industries currently use non-iodized salt for economic reasons and because rarely people may have an allergy to iodine [29,45]. All segments of society should be required to switch to iodized salt. Iodide salts need to be mandated.

Overall, it appears that in the United States the general population has adequate iodine intake but that some pregnant women may be at risk for iodine deficiency. For a population of school-aged children or non-pregnant adults to be iodine sufficient, median urinary iodine concentrations should be greater than 100 mcg/L and no more than 20% of the population should have values lower than 50 mcg/L [46]. During 2007–2008, National Health and Nutrition Examination Survey (NHANES) participants aged 6 years and older had a median urinary iodine concentration of 164 mcg/L and 8.8 ± 0.4% had concentrations lower than 50 mcg/L. Among women of reproductive age, the median urinary iodine concentration in NHANES 2005–2008 was 133 mcg/L and 14.6 ± 1.7% had concentrations lower than 50 mcg/L [47].

### 3.7. Side Effects of KI Prophylaxis

The possible side effects of KI can be reduced by utilizing the lowest effective prophylactic dose. When used by public health authorities for ^131^I exposure, the benefits from thyroid blocking using KI tablets far exceed the health risks for all age groups [13]. The adverse health effects of KI prophylaxis can include mild allergic reactions, such as skin rash or gastrointestinal discomfort [13]. In a retrospective analysis of the effectiveness of KI prophylaxes in Poland following the Chernobyl nuclear incident, minimal side effects were determined for both adults and children [6,11]. Specifically, 2.4% of children and 0.85% adults experienced vomiting; 1.1% children and 1.2% adults developed skin rashes and 0.36% of children and 0.63% of adults experienced abdominal discomfort [6]. These minimal side-effects of KI administration promote prophylaxis usage and a favorable risk/benefit analysis.

Iodine-induced hypothyroidism is a primary side-effect concern in neonates, as dosages 2 to 6 times the normal iodine intake can induce this side-effect [17]. The high sensitivity to both radioiodine exposure as well as excessive stable iodine intake makes the calculation for appropriate dosing in neonates and young children very difficult [17]. 

Patients who have had their thyroid removed or who are hypothyroid for various reasons and are taking thyroid hormone will not need to take KI prophylaxis since their thyroid glands will not concentrate a significant amount of ^131^I (Table A1). Patients with Hashimoto’s thyroiditis who are not taking thyroid hormone may have transient TSH elevation following KI but are unlikely to become symptomatic. Patients with nodular goiter or subclinical Graves’ disease may have transient hyperthyroidism which will occasionally be symptomatic (Table A2). People that should not take KI include those hypersensitive to iodine, those with dermatitis herpetiformis, individuals with hypocomplementemic vasculitis or myotonia congenital—all of these listed conditions are very rare disorders or conditions [13]. Individuals over 40 years of age are less likely to develop thyroid cancer following ^131^I exposure [48] and have a greater chance of developing side-effects [48]. KI prophylaxis is only recommended by the FDA for adults over 40 years with predicted thyroid gland exposure of ≥500 cGy.

## 4. KI Distribution Review

### 4.1. KI Pre-Distribution and Radiological Plumes

In terms of the distribution radius, the ATA has suggested KI pre-distribution from 0 to 50 miles surrounding a nuclear power plant, stockpiling for distribution up to 200 miles, and availability from the National Stockpile at distances greater than 200 miles [9,10]. Evidence is accumulating whereby plumes have been known to spread out greater than a 310 miles radius [49]. It took less than three days following the release of radioactive materials of Fukushima Dai-ichi for the radioactive plume to reach Chiba City, a neighboring city 220 miles south of the plant location [50]. This case indicates not only the distance to which radioactive plumes can migrate, greater than 220 miles, but also, the speed with which such plumes are able to migrate, up to 50 miles daily. This data supports the ATA recommendations for KI availability to the general public at greater than 200 miles surrounding a nuclear power plant. In a 2008 letter to the United States Office of Science and Technology Policy, ATA Chief Operating Officer Richard Kloos expressed that following the Chernobyl nuclear accident, nations within the Chernobyl radiological plume pathway experienced thyroid cancer incidence increases 200 miles from the accident’s source [9,14].

### 4.2. Meteorological Conditions and Plume Size

The plume of a radiological incident is defined as the initial cloud of concentrated radioactive particles released from a NPP during an accident [51]. Plume size is directly related to wind speed and direction, wind turbulence resulting from solar heating, humidity and temperature, and the method of release [51]. Plumes can thus remain relatively local or spread considerable distances depending on the meteorological conditions. The concentration of radiological contaminates and the radiation dose decreases as the plume expands [51]. After the Fukushima Dai-ichi nuclear accident, it was observed that the original plume extends over 300 miles and after further dispersion very small amounts of ^131^I could be detected worldwide [52]. The Chernobyl accident saw radiological plumes travel more than 300 m from the source of the accident [49,53]. Beyond certain threshold distances, radiation doses from plumes will no longer exert significant health effects. For example, only up to 300 miles from the Chernobyl accident site were there increases in the incidence of thyroid cancer in children [14]. There will be variability in the threshold distances radiological plumes exert adverse health effects dependent on meteorological conditions, methods of release and duration of plume travel. Tracking radiological plumes is useful for determining specific at-risk regions to aid and assess, but is limited due to a reduction in health effects at increasing distances.

Since the plume size and migration pattern is greatly affected by wind, an analysis of annual average wind speeds for regions of the United States provides a rough estimate of 24 h radiological plume dispersion potentials. The continental wide average wind speed at altitude of 80 miles falls between 8 and 23.5 miles/h [54]. Therefore, in a 24 h period radioactive plumes can disperse anywhere in the continental United States between 192 to 564 m, assuming all other factors, such as wind direction or wind speed, remain constant. While a plume traveling 500+ miles in a single day is unlikely, the theoretical possibility should encourage attention and implies radiological danger exists at distances potentially overlooked. Meteorological data will have to be accumulated at the time of the accident.

### 4.3. Iodine 131 Dispersal Methods

The main sources of ^131^I release are from medical devices, nuclear testing sites, and nuclear power plants [22]. Cracks, holes or other fenestrations in nuclear reactors cause leakage of ^131^I, while, nuclear explosions can disperse ^131^I rapidly and, as discussed above, to great distances. The heat associated with the immediate explosion can force ^131^I up into the atmosphere at distances potentially exceeding ten kilometers, creating a radioactive plume which is dispersed in the direction of the prevailing wind [22]. ^131^I unites with airborne particulates which either drift to earth or are brought down by precipitation [22]. Subsequent contamination from ^131^I accounts for some of the continued radioiodine exposure following a nuclear event, with contamination in water-sources, flora, and milk produced from livestock. 

While ^131^I was significantly taken up by the thyroid glands of children after Chernobyl from the ingestion of contaminated foodstuffs and milk [53], early control of the dietary pathway in radiological response plans has substantially reduced risk from this exposure pathway. The major route of ^131^I internalization in the United States would almost certainly be from gaseous and particulate ^131^I in the radioactive plume [17]. Gaseous molecular iodine and gaseous particulate iodine are completely absorbed through the respiratory and upper digestive tract and occurs rapidly (10 min half-life) [17,55,56]. Pulmonary retention of organic gaseous iodide is ~70% and also occurs rapidly (5 s half-life) [17,57]. 

### 4.4. Iodine 131 Historical Radiological Exposures

Historical ^131^I releases include: the Marshall Islands Nuclear Testing Site where 6,300,000,000Ci or 233,100 PBq of ^131^I was released between 1946 and 1958, the Nevada Nuclear Test Site where 150,000,000 Ci or 5,550 PBq of ^131^I was released between 1952 and 1970, Chernobyl where 50,000,000 Ci ^131^I or 1,850 PBq was released in 1986, and the Hanford Reservation Nuclear Production Complex in Washington where 740,000 Ci ^131^I or 27.38 PBq was released between 1944 and 1972 [22,58]. Clarification of radiation units and measurements are seen in Table A3 and Table A4.

The Chernobyl nuclear accident [22] resulted in nearly 5,000 thyroid cancers in children [59]—a 100-fold increase in the incidence of thyroid cancer in children [9]. Mild estimations are that a nuclear incident in the United States 1/20th of the magnitude of Chernobyl could create a 5-fold increase in the incidence of thyroid cancer in iodine-replete American children [9].

An estimated 511 PBq (1.38 × 10^7^ Ci) of ^131^I was released from the Fukushima nuclear accident since 15–16 March 2011 [60]. There are 23 nuclear reactors with the same design as the Fukushima Dai-ichi nuclear power plant operating in the United States [9]. Small amounts of ^131^I dispersed from Fukushima have been detected in North America and Europe [61]. The Fukushima Dai-ichi nuclear power plant accident is an example of a contemporary nuclear plant accident with serious implications, with radiological plumes extending greater than 200 m and very high levels of local ^131^I release. 

Following the 2011 Fukushima Dai-ichi NPP accident, several decisions were made in the emergency response reaction by the Japanese government and Tokyo Electric Power Company Corporation (TEPCO) that should be assessed for future radiological emergency plans. The Japanese Central government and TEPCO, the primary corporation responsible for the cleanup and maintenance of the Fukushima NPP accident, did not release early radiation maps to the public [62]. This decision may have hindered local government’s evacuation route planning, as some evacuation routes led through the primary contaminated area [62]. Furthermore, the Japanese government, in response to the Fukushima accident, raised the allowable radiation exposure levels in children to 20 times higher than historic precedents [63]. The decision may have been made to reduce the psychological effects caused by fear and phobias of radiation exposure. The Japanese government advised sheltering, prompt evacuation for at-risk contaminated regions and control of the dietary pathway [64]. 

In regard to KI prophylaxis, TEPCO utilized 17,500 KI tablets for 2,000 onsite workers, on average 20 tablets per individual, with one individual receiving and taking 85 tablets [65]. The Japanese government distributed iodine pills to children in contaminated regions but not the general public [64,66]. The thyroidal exposure dose before which children are advised to take iodine tablets is 100 mSv in Japan [64]. Several weeks following the accident, children from the most contaminated regions (*n* = 1,080) were monitored for radiation dose, none of which experienced the thyroidal dose necessary for iodine tablet recommendation [64]. Tangen *et al.* does mention that Norwegian standards of projected thyroid dose differ from Japanese, in which as little as 10 mSv would merit iodine distribution [65]. Under these guidelines, some of the monitored children qualified for iodine prophylaxis [65]. 

The Fukushima NPP accident has had health implications due to the high levels of radiation released and vast area over which the radiation has dispersed—with an initial estimate of 900,000 TBq released, in contrast to 5,200,000 TBq released in Chernobyl [67]. The significant radiation release, as likened to Chernobyl, reflects the context and severity of the Fukushima accident. Most of the radiation fell into the Pacific Ocean; however, 19% of the radiation was deposited over land [68]. The level of ^137^Cs that was released is likened to Chernobyl levels, with 100,000 TBq released [69]. Furthermore, the levels of radioactive cesium measured off the coast of Japan peaked at more than 100,000 Bq per cubic meter in early April, 2011, significantly affecting the fish market and associated industries [63]. 

In order to study the health effects of the Fukushima reactor accident Fukushima University initiated the Fukushima Health Management Survey. As part of this survey thyroid ultrasound examinations were to be performed on all 360,000 children from the region who were between ages 0 and 18 years at the time of the accident within 3 years, and then annually [70]. As of November 2013 254,280 children had been screened. 1.2% had thyroid nodules: 0.7 % had nodules that were <5 mm and 0.5% had nodules that were >5 mm. 46.4% had cysts that were <20 mm and <0.01% had cysts that were >20 mm [70]. A control group of 4,365 children between 3 and 18 were studied in three prefectures, which were geographically separated and thought to be unaffected by radioactive material from the Fukushima accident. In this control group 56.6% of children tested had cysts and 1.6% had thyroid nodules: 4.6% had cysts greater than 5 mm and 1.0% had nodules greater than 5 mm [71]. Based upon this data, the level of thyroid abnormalities found in the Fukushima children may be normal for Japanese children aged 18 and under. 

Reported thyroid cancer rates in Japan have increased significantly since the 2011 Fukushima nuclear event [72]. However the increased reported rates of thyroid cancer can be attributed to discovery of pre-existing thyroid cancers detected by intense screening and should not be confused as resulting directly from the Fukushima nuclear incident. Following extensive thyroid ultrasound screening of 254,280 Fukushima children, there have been 32 confirmed and 43 suspected thyroid cancer cases in children as of November 2013. Only four of the cancer patients were < 11 years old, and the youngest was 6 years old. [70]. The 13 per 100,000 incidence of confirmed thyroid cancer cases in children contrasts with 2007 thyroid cancer incidence rates in children of 0.1 per 100,000 below age 15 and 0.9 per 100,000 for ages 15 to 19 years [72,73]. However thyroid cancer is a very slowly growing tumor. Without intense screening, the majority of these cancers would not have been detected until adolescence or adulthood—or perhaps not at all. In the control population, 17.9% of adult Japanese who were not exposed to radiation at Hiroshima or Nagasaki were found to have papillary thyroid cancers at autopsy [74]. Furthermore, after Chernobyl, which resulted in a greater release of ^131^I, and after which the local population was not evacuated, thyroid cancer was first detected 4–5 years after the accident and only later in adolescents. It is also possible that the high iodine content of the Japanese diet may have some protective effect as compared to Chernobyl which is in an area of moderate iodine deficiency. Any adverse effects to the thyroid are thus not likely to become manifest in for several more years. The present thyroid ultrasound results will serve as highly valuable controls for follow-up screenings.

The Nevada Nuclear Test Site has had some of the greatest historical amounts of ^131^I released on land, with 150,000,000Ci ^131^I released between 1952 and 1970 [22]. The Nevada Test Site had over ninety different tests, accounting for 99% of all ^131^I release between 1952 and 1957 [22]. Extensive studies of fallout from these tests mandated by congress and carried out by the National Cancer Institute (NCI) showed that fallout affected virtually every part of the continental US [75]. This site was the main location of above ground nuclear bomb detonation tests for the USA in the 1950s [76]. In 1997 the NCI announced the initial results of a study of fallout from the atmospheric nuclear bomb tests conducted at the Nevada test site from 1951 to 1958. They found the cumulative average radiation dose to the thyroid gland to be 1 to 4 rad but as much as 16 rad in high-deposition areas. As a worst case scenario they estimated that between 10,000 and 75,000 children in the US might develop thyroid cancer as a result [77]. In a 2010 publication the NCI calculated the ERR of developing thyroid cancer to be 1.8 (95% confidence interval (CI), 0.5–3.2) for every Gray (one Gray = 100 rad) of absorbed radiation, but only for exposure before one year of age. The ERR is an epidemiological risk measure that quantifies how much the level of risk among persons with a given level of exposure exceeds the risk of non-exposed persons [78]; in this case, it is 80% greater. They conclude that “the study adds support for an increased risk of thyroid cancer due to fallout, although the data are inadequate to quantitate it” [79]. These historical precedents provide impetus for concern about the potential for future accidents, Table 4 is a list of historical releases of ^131^I [80]. 

**Table 4:** Iodine 131 historic releases.

Total Estimated Amount of ^131^I Released from the Site (Ci)	Total Estimated ^131^I Release from Site (PBq)	Site of Event	Time Period
6,300,000,000	233,100	Marshall Islands Nuclear Testing Program	1946–1958
150,000,000	5,550	Nevada Test Site, Nevada	1952–1970
50,000,000	1,850	Chernobyl (former Soviet Union)	1986
13,800,000	511	Fukushima	2011
740,000	27.38	Hanford Reservation, Washington	1944–1972
60,000	2.22	Savannah River Site, South Carolina	1955–1990
8,000 – 42,000	0.296 – 1.554	Oak Ridge National Laboratory, Tennessee	1944–1956
20,000	0.74	Windscale, United Kingdom	1957
15 – 21	5.55 × 10^-4^ – 7.77 × 10^-4^	Three Mile Island, Pennsylvania	1979

*Source*: modified from United States Department of Health and Human Services; Ten Hoeve and Jacobson; Plasman; Tokyo Electric Power Company [22,58,60,68].

## 5. Iodine 131 Physiology, Health, and Therapeutics

### 5.1. Iodine 131 Routes to Exposure

Radiation is a form of energy that we are exposed to in small amounts every day. Ultraviolet (UV) radiation from sunlight is relatively weak and cannot penetrate the skin. However, overexposure can cause sunburn and eventually skin cancer. More powerful ionizing radiation comes from background radiation, cosmic radiation and medical exposure through X-rays, CT scans *etc*. This form of radiation can cause mutations and cancer of internal organs. Average ionizing radiation exposure is primarily from natural sources (82%) yet also results from human activities (18%) [81]. Actual annual radiation exposure is variable, dependent on lifestyle choices, but has been summarized by Fentiman *et al.* who claims that 55% of annual ionizing radiation exposure results from radon (200 mrem/year), 11% from human internal processes (40 mrem/year), 8% from the soil and rocks (28 mrem/year), 8% from cosmic radiation (27 mrem/year) and 18% from human activities: such as X-ray scans (39 mrem/year), nuclear medicine (14 mrem/year) and other sources (10 mrem/year) [81]. 

Radiation emergencies are usually caused by man-made radiation sources, such as, NPP accidents or nuclear bomb detonations. These emergencies involve exposure to unwanted ionizing radiation and contamination by radionucleotides which can be internalized and in some instances remains in the body indefinitely. Iodine-131 also has many industrial and medical uses, which increase opportunities for ^131^I exposure and contamination. 

### 5.2. Iodine 131 Field Detection Methods

There are currently four principal types of radiation detection devices in use, the personal radiation detector (PRD); the handheld survey meter (HSM), the radiation isotope identification device (RIID), and the radiation portal monitor (RPM) [82]. The PRD is a wearable gamma and/or neutron radiation detector, which numerically displays radiation intensity (on a scale of 0–9) but cannot specify the type, of radioactive source [82]. The HSM acts similarly to the PRD measuring the amount of radiation present; it can be used from greater distances than the PRD but also cannot specify the type, of radioactive source [82]. The RIID, however, can be used to identify the specific radioactive material present by analyzing the energy spectrum of radiation [82]. These instruments provide real-time analysis of radioactive materials [82]. The RPM is a large radiation monitor portal, through which trucks, vehicles, ships, or other large transporting vessel can pass in real time, which identifies radioactive material [82]. Although the technology readily exists and can substantially improve response times in the event of a radiological emergency, for example, there are currently no real-time radioactive iodine monitoring systems utilized in the NPPs of New York State [83].

### 5.3. Radiation Phobias and Psychological Effects

Results from studies on the psychological effects due to nuclear accidents found that residents of near NPP accidents, such as Three Mile Island, had increased anxiety, a heightened perception of risk, subclinical depression and demoralization for six years following the event that did not return to normal until ten years after the incident [22]. Mental health has been implicated as the greatest issue with the Fukushima Dai-ichi nuclear incident, even greater than the immediate physical effects due to radiation exposure. The stress of dislocation, uncertainty, and worry about invisible radiation is linked to physical issues, such as heart disease. Stressors also contribute to the development of unhealthy lifestyle choices, such as poor dietary choices, lack of exercise and sleep deprivation, which all lead to additional physical ailments [84]. An investigation by Evelyn Bromet of the outcomes from NPP accidents determined that the most at-risk individuals, emotionally, of nuclear accidents were the clean-up workers and mothers of young children [85]. Preliminary evidence following the Fukushima Dai-ichi nuclear accident has indicated that cleanup workers and mothers of young children risk various psychological ailments: including anxiety, depression, post-traumatic stress and psychosomatic problems [85]. These psychological effects and resultant physical detriments can be reduced significantly by the development and implementation of strong emergency preparedness plans. 

### 5.4. Federal and State Emergency Preparedness Plans

On 12 June 2002 President George W. Bush signed the Public Health Security and Bioterrorism Preparedness and Response Act of 2002 (Bioterrorism Act). Section 127 of this Act required states to consider using KI in their emergency preparedness plans and offered States, with the successful submission of a plan to stockpile KI tablets and for use in the event of a nuclear accident, a quantity of KI tablets sufficient for distribution to the general public living within 20 miles of a nuclear power plant [7]. Section 127 of the Bioterrorism Act had a back-out clause (f), which stated, if an alternative, more effective prophylaxis or preventative measure is determined and utilized, Section 127 would cease to apply [8]. On 22 January 2008, after having been delegated the authority to invoke the waiver by the President, John H. Marburger III, Director of the Office of Science and Technology Policy, invoked the waiver, determining that avoidance of exposure altogether through interdiction of contaminated food and evacuation of the potentially affected population was a better preventative measure than KI usage [8]. This decision did not take into consideration the need for prophylaxis within two hours of exposure, like in the case of certain natural disasters or terror events, where evacuation is impossible. Events in which evacuation is impossible can also be the cause of nuclear accidents, as was the case in Fukushima, where some residents were unable to evacuate due to damage caused by the earthquake and resultant tsunami. Therefore, we propose that the federal government should amend recommendations for radiological emergencies to recognize once again the necessity to pre-distribute KI tablets to the at-risk population for nuclear accidents. Many states are continuing to employ KI in their emergency preparedness plans for radiological emergencies. Usage of KI and emergency preparedness, covering WHO, USNRC, ATA, FDA, CDC, Federal Emergency Management Agency (FEMA), NYS, USA, and New Jersey State (NJS) recommendations are reviewed in Table 5. Current recommendations of governmental agencies are inadequate to protect against radionuclear emergencies and do not address the problem of iodine deficiency and public health. 

Since the Bioterrorism Act, states like NYS have actively revised their emergency preparedness plan to incorporate the FDA recommendations for KI use for thyroid prophylaxis in the event of radioiodine exposure and endorse the levels of KI use as suggested by the FDA [86]. The NYS radiological emergency preparedness plan stresses that the overall benefits of KI far exceed the risks of overdosing, especially for children, and stresses the importance of pre-distribution [86]. While the pre-distribution efforts of states which have adopted KI into their emergency preparedness plans are of the appropriate intention, the effectiveness of pre-distribution methods needs further evaluation. Currently, pre-distribution to the general public is near zero, for example, NJS has only pre-distributed to ~10% of people within the 10-mile Emergency Planning Zone (EPZ) and NYS 15% within the 10 m EPZ as of a 2004. 

A National Academy of Sciences study determined the effectiveness of distribution and administration of KI in the event of a nuclear incident [87]. NYS pre-distribution is limited to a single dose of KI to individuals or a single liquid bottle of KI to a family [88]. NYS recommends that the quantity of KI tablets pre-distributed be equal to the evacuation time estimate (ETE), in days rounded up, times one age and weight dependent dose/day [88]. For the four operating NYS nuclear power plants, including the Ginna Nuclear Generating Station, Indian Point Energy Center, James A FitzPatrick Nuclear Power Plant and Nine Mile Point Nuclear Generating Station, the general population ETEs range from 1:30 (h:min) to 5:25 at the 90th percentile [89,90,91]. Since these evacuation times are less than a single day, people are only pre-distributed a single KI dose. Further doses will be made available in a radiological event from key distribution locations, insinuating that one day is ample time for evacuation and/or the acquiring of additional KI doses [86]. This plan needs to be revised as evacuation is sometimes impossible. 

**Table 5:** Governmental body/agency, KI usage and emergency preparedness recommendations.

Governmental Body/Agency	Distribution Recommended	Pre-Distribution Recommended	Pre-Distribution Effectiveness	Pre-Distribution Distance	KI Dose
United States of America	Yes	Yes	N/A	20 miles	FDA-Endorse
New Jersey State	Yes	Yes at public education & Distribution sessions	~10%	10 miles EPZ	FDA-Endorse
New York State	Yes	Distribution by county; Pick-up locations; Via mail	15% in EPZ	Offered KI regardless of distance	FDA-Endorse
World Health Organization	Yes	Yes	N/A	N/A	N/A
United States Nuclear Regulatory Commission	Yes	Yes	N/A	10 miles radius	FDA-Endorse
American Thyroid Association	Yes	Yes	N/A	50 miles Pre-Distribution; 50–200 miles Stockpile Local Public Facilities; >200 miles National stockpile	FDA-Endorse
Food and Drug Agency	Yes	Yes	N/A	10 miles radius of USNRC mentioned	
Centers for Disease Control and Prevention	N/A	N/A	N/A	Public health or Emergency managers to decide	FDA-Endorse
Federal Emergenct Management Agency	Yes	Yes	N/A	10 miles radius	FDA-Endorse

Notes: EPZ—Emergency Planning Zone; N/A—Not Applicable; FDA-Endorse—The governmental body/agency endorses the FDA’s recommendations for KI dosage.

Since the 2004 pre-distribution efforts, all tablets and liquid formulas of KI have since expired, having shelf-lives of 5–7 years. Furthermore, past pre-distributions do not reflect peoples who have moved to EPZ areas in recent years, which would reduce the number of individuals with KI.

An in-depth review of the NYS radiological emergency preparedness plan also indicates weaknesses in the response time to emergencies. According to the NYS radiological emergency preparedness plan, it takes anywhere from 150–210 min after a General Emergency has been declared to inform the public to take KI [86]. This response time is too slow for citizens, in the immediate proximity of the NPP because the effectiveness of KI, ^131^I blockage, is reduced by 50% in two hours that follow radioiodine release [86]. Real-time monitoring of radioiodine levels can reduce the time it takes to inform the public. The United States government has numerous ways to check for reactor containment breaches, yet currently no NYS NPP utilizes real-time iodine monitoring [86]. Real-time iodine monitoring could help in determining the radioactive iodine profile of the accident and in calculating ^131^I projected radiation doses so appropriate countermeasures can be taken.

A final and important question which requires further investigation is how the public will be notified about a nuclear incident. Having KI is of no benefit unless it is taken in time. One proposed system would utilize the existing severe weather emergency warning system. Arrangements should be made to incorporate warnings about nuclear accidents into this system. Potassium Iodide pre-distribution with clear instructions for use and highly efficient warning methods are thus paramount to successful radioiodine prophylaxis for populations located in close-proximity to the nuclear reactors.

### 5.5. Iodine 131 Industrial Usage

Even in the absence of nuclear testing or nuclear accidents, we are exposed to very minute amounts of ^131^I. Iodine 131 for industrial and medical use is a reactor byproduct produced during the fission of Uranium with a 2.878% yield [1]. In 2013, globally, there were 428 operating nuclear power plants; Table 6 lists the number of nuclear reactors in operation by nation in 2013. Since 1951, ^131^I has been the gamma emitting radioactive-tracer most used in leak detection and isotope hydrology [92,93,94,95]. Currently ^131^I is being employed in hydraulic fracturing for the US oil industry, to provide information regarding flows and underground leaks [96,97]. This industrial use can cause contamination of groundwater sources with the potential for detrimental health results. The medical uses of ^131^I are discussed in section 5.6. The availability of ^131^I from these sources creates a potential opportunity for a terrorist act.

**Table 6:** Nuclear reactors in operation worldwide in 2013.

United States of America	100	Sweden	10	Bulgaria	2
France	58	Germany	9	South Africa	2
Japan	50	Spain	7	Romania	2
Russia	34	Belgium	7	Mexico	2
Republic of Korea	23	Chech Republic	6	Argentina	2
India	20	Switzerland	5	Brazil	2
Canada	19	Hungary	4	Netherlands	1
China	17	Slovakia	4	Armenia	1
United Kingdom	17	Finland	4	Slovenia	1
Ukraine	15	Pakistan	3	Iran	1

*Source*: Statista.com [98].

### 5.6. Health Effects and Medical/Therapeutic Use

While we have recognized ^131^I as being utilized in many industrial practices, ^131^I also has many medical, diagnostic and therapeutic applications. Since 1941, as documented in a Saul Hertz report [99] ^131^I has been used medically to induce tissue destruction of the thyroid, remedying hyperthyroidism, iodine absorptive thyroid cancer, and thyroid nodules with hyperactivity [99,100]. In diagnostics, ^131^I can be used as a radioactive label for molecules that localize in various benign or malignant tissues known as radiopharmaceuticals [99]. After injection they can be localized by scanning the body, providing useful diagnostic information [99].

We have come to learn a great deal about the health effects of ^131^I exposure, resultant co-morbidities, and best-practices for reducing ^131^I exposure from medical, therapeutic and diagnostic, practices; See Table 7. 

**Table 7:** The radiation dosimetry of ^131^I administration to a euthyroid patient average weight (70 kg) in both milligray (mGy) per megabecquerel (MBq) and rad per millicurie (mCi).

Tissue	Thyroid Uptake					
	5%		15%		25%	
	mGy/MBq	Rads/mCi	mGy/MBq	Rads/mCi	mGy/MBq	Rads/mCi
Thyroid	72	266	210	777	360	1300
Stomach Wall	0.45	1.7	0.46	1.7	0.46	1.7
Red Marrow	0.038	0.14	0.054	0.20	0.07	0.26
Liver	0.03	0.11	0.032	0.12	0.035	0.13
Testes	0.029	0.11	0.028	0.10	0.027	0.10
Ovaries	0.044	0.16	0.043	0.16	0.043	0.16
Urinary Bladder	0.58	2.1	0.52	1.9	0.46	1.7
Salivary Glands	0.5	1.85	0.5	1.85	0.5	1.85
Other	0.040	0.15	0.065	0.24	0.090	0.33

Note: MBq and mCi are measures of the quantity of radioactive material present based on the number of disintegrations per sec; while mGy and rad are used to quantitate the amount of radiation absorbed by an organ. Source: Mallinckrodt Inc. Drug Information Online *Sodium Iodide I 131* [100].

### 5.7. Iodine 131 Medical Dosage and Public Legal Limit

The legal limit for the general public to possess radioactive iodine is 37 kBq (1µ Ci) ^131^I [101], and some administrative kits used by radiopharmacies to create therapeutic dosages, Hicon specifically, contain up to 37,000 MBq (1,000 mCi) ^131^I [102]. The dose range of sodium ^131^I for hyperthyroidism treatments range from 148 to 370 MBq (4 to 10 mCi), and higher dosages are used to treat toxic nodular goiter or other unique conditions [100]. For the treatment of thyroid carcinoma, therapeutic doses of ^131^I can range from 3,700 MBq to as high as 9,250 MBq (100–250 mCi) [100,101,103,104]. 

### 5.8. Iodine-131 Exposure Side Effects and Condition Co-Morbidities

Iodine 131 should never be given to a pregnant woman for any reason. If there is any doubt a pregnancy test should be performed. The fetal thyroid starts to concentrate iodine at about 12 weeks gestation and due to its small size radiation doses are much higher than in children or adults. Large radiation doses destroy the gland resulting in in-utero hypothyroidism which if untreated causes severe mental retardation, while smaller doses increase the risk of thyroid cancer.

Side effects of treatment with ^131^I are dose-related. They are rare with the relatively low doses used to treat hyperthyroidism but increase significantly when thyroid cancer patients are treated with more than 3.7 GBq (100 mCi). The most severe permanent side effects occur in thyroid cancer patients with metastatic disease who have been treated multiple times [105,106]. Dose related side effects have been reported and are listed in Table 8 [106,107,108]. A study from the pre-ultrasound era, determined that the use of ^131^I therapy for the treatment of hyperthyroidism in Graves disease was associated with an increased risk of benign thyroid neoplasms in children [109]. 

**Table 8:** Adverse Reactions to high dose ^131^I treatment.

Severity	Conditions
**Transient (high dose)**	Radiation sickness (nausea, vomiting, fatigue)
	Hypersensitivity or allergic reactions (extremely rare)
	Sialadenitis (pain and swelling of salivary glands)
	Loss of taste
	Radiation-induced thyroiditis (swelling and tenderness in the neck)
	**Bone marrow suppression (platelet count most sensitive)**
	Decreased sperm count (can last up to 6 months)
	Chromosomal abnormalities in circulating lymphocytes
**Permanent (high dose)**	Dry mouth (due to decreased production of saliva)
	Excessive tearing (due to fibrosis of tear ducts)
	Infertility – rare even with very large cumulative doses of ^131^I
	**Bone marrow suppression (anemia, leukopenia, thrombocytopenia)**
	**Pulmonary fibrosis (only in patients with diffuse lung uptake)**
	**Leukemia and other secondary cancers**

The most serious effects are in bold letters. *Source*: modified from Mallinckrodt Inc. Drug Information Online; Silberstein *et al*. [100,110].

High dose ^131^I for treatment of thyroid cancer has been implicated in inducing leukemia and other secondary malignancies. The studies up to 2008 are reviewed by Dr. Elaine Ron from the NCI, who has published extensively on this subject in the past [111]. In 2008 a meta-analysis by Sawaka *et al*., compared the relative risk of secondary malignancy in thyroid cancer patients treated with ^131^I with those who were not treated with ^131^I. The relative risk for any secondary malignancy was 1.19 (95% confidence limits 1.04–1.36). For leukemia the relative risk was 2.5 (95% confidence limits 1.13–5.53), but relative risks for other specific organs did not reach significance [112]. 

Radiation has also been implicated as increasing the incidence and prevalence of thyroid nodules; this was clearly demonstrated in the Chernobyl reactor accident in 1986. The accident was associated with a 100-fold increase in the incidence of thyroid cancer amongst children, beginning 4 years after the incident, in radioiodine-contaminated regions, namely Belarus, Ukraine, and the Russian Federation, where radiation exposures were ≥5 cGy [113,114,115]. The use of KI prophylaxis in Poland that is approximately 400 m from Chernobyl reduced a rise in radiation-related thyroid cancer incidence [6,11]. The KI was overall well tolerated. Side effects reported included gastrointestinal distress and rash. Of note, the newborns receiving single doses of 15 mg of KI showed transient hypothyroidism [15]. 

### 5.9. Post Iodine 131 Exposure Patient Precautions

After ingestion of ^131^I some is concentrated in the thyroid and the remainder is excreted within 3–5 days, predominantly via the urine with smaller amounts in stool or perspiration. Most of the ^131^I in the thyroid decays there, but a small amount is released back into the circulation as ^131^I labeled thyroid hormones. These are deiodinated in peripheral tissues releasing ^131^I that in turn can either re-enter the thyroid or be excreted. Patients who have been given large amounts of ^131^I as treatment for hyperthyroidism or thyroid cancer are advised to not have sexual intercourse for the first month and women are warned to avoid pregnancy for six months, in order to reduce exposure of sperm or the fetus to residual radioiodine [99]. Women must stop breast-feeding several months prior to ^131^I therapy and may not resume afterwards since the lactating breast concentrates significant amounts of ^131^I [110].

These post treatment precautions have been carefully determined by healthcare personnel and governmental agencies, for the reduction of radioiodine exposures following hospital release of ^131^I therapy patients [116]. The amounts of ^131^I used for the treatment of thyroid disease are orders of magnitude greater than the public is likely to be exposed to in the course of a reactor accident, and such strict precautions are not necessary with one exception. Because the thyroid glands of infants are exquisitely sensitive to radiation, mothers should stop nursing for several days after exposure to ^131^I.

## 6. Future Perspectives

### 6.1. Iodine 131 Potential in a Dirty Bomb

The ATA has suggested that due to the short half-life of radioactive iodine, a dirty bomb is unlikely to contain ^131^I. They state that health authorities will determine its presence in such an instance and will react accordingly [117]. The CDC states that taking KI in the event of a dirty bomb will probably not be beneficial; furthermore, it indicates that KI can be dangerous to some people [118]. 

Upon careful review of this literature, we encourage the CDC and consequently the ATA to revise their advice regarding KI use in response to a dirty bomb. In the event of a dirty bomb, it is hard to measure the immediate presence of ^131^I. In order to measure ^131^I presence in a dirty bomb, a RIID that can analyze the energy spectrum of radiation is needed [82]. This handheld device currently costs tens of thousands of dollars, and so, would only be available to limited groups of people. While the RIID device does provide real-time monitoring of radioactive isotopes, there can be a major lag time between a dirty bomb event and the identification of radioactive isotopes, while those in the direct proximity of the dirty bomb are affected immediately. Potassium iodide blockage of ^131^I three to four hours after radioiodine reduces exposure by 50%, so the prompt administration of KI is paramount [83]. First responders should be equipped with KI for prophylactic administration in such instances. Break-ins at medical manufacturing facilities provide opportunities for ^131^I access, which could be used to create dirty bombs. The short half-life of ^131^I limits its radioactive potential as a dirty bomb; however, its capacity to induce terror remains high. This makes ^131^I a likely source for use in a dirty bomb. 

### 6.2. Worldwide Iodine Deficiency

In a 2006 review, of 130 participating countries iodine deficiency disorder (IDD) was a public health concern in 47 countries that comprised 91.1% of the worldwide population [98]. IDD is the world’s leading cause of preventable brain damage, inducing average reductions in IQ of 10 to 15 points. Worldwide, 266 million school-aged children are estimated to have insufficient iodine intake, inducing a myriad of learning disabilities and psychomotor impairments [119,120,121,122]. Iodine deficient children and pregnant women have higher thyroidal uptakes of ^131^I (Table A5 and Table A6) and are therefore especially vulnerable to the effects of ^131^I contamination [120]. In some instances young adults may be more at risk of developing mild iodine deficiency than younger children due to a tendency to decrease their dairy intake [122]. The number of nations with iodine deficient children has decreased from 54 to 30 in the last ten years [123]. This developing trend provides optimism for worldwide iodine sufficiency. Table 9 provides a current review of worldwide iodine deficiency.

**Table 9:** Worldwide Iodine Deficiency in Urinary Concentrations.

Moderate Iodine Deficient Nations	Mild Iodine Deficient Nations
(Median Urinary Iodine Concentrations 20–49 μg/L)	(Median Urinary Iodine Moderate, 50–99μg/L)
Afghanistan	Albania
Algeria	Burundi
Central African Republic	Democratic People’s Republic of Korea
Ethiopia	Estonia
Gambia	Finland
Ghana	Guatemala
Papua New Guinea	Haiti
Vanuatu	Hungary
	Ireland
	Italy
	Lebanon
	Lithuania
	Mali
	Morocco
	Mozambique
	New Zealand
	Russian Federation
	Sudan
	Ukraine
	United Kingdom

*Source*: Modified from Zimmermann, M.B. [123].

Although global iodine deficiency trends are optimistic, iodide insufficiency may be more widespread than previously thought [124,125,126,127], however, additional research is required to provide a reasonable consensus. A few studies show that KI is effective in treating benign solitary solid cold nodules of the thyroid and suppressing nodules of the thyroid [126,127,128,129]. However, no one uses KI to treat thyroid nodules because to be effective TSH must be suppressed which has side effects including atrial fibrillation. For completeness Table 10 lists the recommended iodine dietary intake dependent on life stage.

Standard text suggests that the thyroid must absorb ~60 mcg iodine daily for normal thyroid hormone production. Iodine insufficiency is associated with cretinism, mental deficiency, lower IQ by 10 to 15 points, hypothyroidism, and a high national presence of thyroid nodules [119,120,121,122]. 

**Table 10:** Life-Stage Recommended Dietary Iodine Intake (Likely Underestimated).

Life Stage (Age)	Recommended Daily Dietary Iodine Allowance
Infancy (Birth to 6 months)	110 mcg
Infancy (7 to 12 months)	130 mcg
Childhood (1 to 8 years)	90 mcg
Preteen (9 to 13 years)	120 mcg
Teens and Adults (14+ years)	150 mcg
Pregnant Adults	220 mcg *
Lactacting Adults	290 mcg *

*Source*: Modified from Institute of Medicine, Food and Nutrition Board, Dietary Reference Intakes for Vitamin A, Vitamin K, Arsenic, Boron, Chromium, Copper, Iodine, Iron, Manganese, Molybdenum, Nickel, Silicon, Vanadium, and Zinc [42] * More than adequate iodine status for all adults.

## 7. Our Recommendations Based on This Review

### 7.1. Potassium Iodide Prophylaxis is Effective and Necessary

The thyroid cancer incidence surrounding the Chernobyl nuclear plant increased 100 fold, with around 5,000 cases resulting from the Chernobyl nuclear accident [9,59]. The incidence of thyroid cancer amongst children began 4 years after the Chernobyl nuclear incident. These historical precedents provide impetus for concern about the potential for future accidents. The benefits from thyroid blocking using KI tablets far exceed the health risks [13].

Following the Chernobyl nuclear event Poland was able to reduce the projected radioiodine thyroid dose of individuals given KI within 24 h of the accident by nearly 40% [6] with minimal side effects determined for both adults and children [6,11]. Alimentary sources of iodine, particularly kelp, offer an alternative form of prophylaxis if KI is not available. 

### 7.2. The Effectiveness of KI Prophylaxis is Lost if Administration is Delayed

Potassium iodide resources must be pre-distributed in EPZ and made available via emergency preparedness plans because taken at the time of exposure they are 99% effective, however by 3–4 h following exposure they are reduced to being 50% effective. Additional doses of KI should be distributed to individuals who risk continued exposure; *i.e.*, are unable to evacuate. The population can be advised by radio or TV whether they need to continue KI prophylaxis.

### 7.3. Extension of the Thyroid Disease Risk Area to Exceed 300 Miles

Radioactive plumes from the Chernobyl accident containing ^131^I caused benign and malignant thyroid nodules to develop, especially in children within a 310 miles radius of the incident [14,49]. Plume size is directly related to wind speed and direction, wind turbulence resulting from solar heating, humidity and temperature, and the method of release [51]. The current recommendation is for KI availability to people 200 miles from a NPP. Plume radii for nuclear events have been shown to exceed 300 m [49,53]. Extension of KI availability to 300 miles only further underscores the inadequacy of current preparedness plans. 

### 7.4. Reduce Costs of Stable Iodide Prophylaxis to Extended Thyroid Disease Risk Area

In order to reduce the costs of emergency preparedness without sacrificing effectiveness and make possible the extension of prophylaxis to effected populations within the first two hours of a ^131^I exposure, the lowest effective prophylactic dose should be used. Based on the Sternthal *et al*. [5] study we recommend the following dosage regimen as useful prophylaxis. An initial dose of 2 mg of SI per 10 pounds taken, on the first day of radioiodine exposure and a protracted daily dose of 1 mg SI per 10 pounds, taken based on the risk for continued exposure. Considering that a mg/lb recommendation may be too complicated for routine use, a simplified plan has been created based on 15 mg tabs, see Table 2. 

We recommend investigation of KI granules, which have an extended shelf life of 20 years as compared to the 5–7 year shelf-life of KI tablets. Furthermore, we recommend distribution of KI to be in opaque sealed containers containing a desiccant, thereby effectively mitigating sources of iodine loss. The increased shelf life would reduce the costs associated with restocking KI distribution centers and stockpiles, and would increase the likelihood that families would maintain a KI stock within its expiration date.

### 7.5. Give Information and Clear Instructions

KI pre-distribution with clear instructions for use is important for successful radioiodine prophylaxis. The general public and particularly populations within EPZ need information and instructions about the administration, alternative dietary sources, as well as, side-effects and contraindications of stable iodide. Negative psychological effects can be reduced significantly by transparency about the dangers of radioiodine and nuclear events as a part of the implementation of strong emergency preparedness plans. 

### 7.6. Facilitate Good Nutrition in Populations with Iodine Deficiency

Thyroidal uptake of radioactive iodine is higher in people with iodine deficiency than in people with iodine sufficiency. For this reason, iodine-deficient individuals have a greater risk of developing radiation-induced thyroid cancer when exposed to radioactive iodine [6]. Worldwide, 266 million school-aged children have insufficient iodine intake [121]. Fast food and pre-prepared food industries can utilize iodized salt to help mitigate iodine deficiencies in at-risk regions. Iodination of salt is an effective and inexpensive means of eliminating iodine deficiency [125]. 

### 7.7. Avoidance of Exposure Is No Longer an Adequate Response to a Nuclear Event

Avoidance of exposure through interdiction of contaminated food and evacuation was recommended and was considered a better preventative measure than KI prophylaxis [8]. This assumes adequate warning and rapid evacuation in highly congested urban areas. Events such as tidal wave in which evacuation is impossible can also be a cause of nuclear accidents, as was the case in Fukushima. In no way should KI prophylaxis replace other radiological emergency procedures, such as control of the dietary pathway and evacuation. KI prophylaxis is an effective supplemental technique intended to improve radiological emergency plans. It is critical that educational programs are established that clearly and effectively get this point across. We propose that the federal government should amend recommendations for radiological emergencies to once again recognize the effectiveness and usefulness of KI prophylaxis in instances of ^131^I exposure.

### 7.8. Plan for Swift Access to Radiation Isotope Identification Device (RIID)

RIID’s can analyze the energy spectrum of radiation. Currently, there is no real time radioiodine monitoring in use in New York State NPP, the serious lag in response time to emergencies greatly reducing the effectiveness of KI prophylaxis in such an event [83]. In the event of a dirty bomb, it is difficult to determine the immediate presence of ^131^I and a RIID is needed [82].

## 8. Conclusions

Preventative measures and robust emergency preparedness plans are paramount to a successful emergency response. In the wake of the recent Fukushima Dai-ichi nuclear accident, revision of KI distribution plans and dosage schemes need to be considered. 

Iodine 131 ingested or inhaled after a reactor accident and concentrated in the thyroid gland can damage thyroid cells causing mutations that eventually lead to thyroid cancer. Young children and developing fetuses are particularly vulnerable [22]. Therefore, we recommend stable iodine prophylaxis in the event of another reactor incident in the United States and globally. An initial dose of approximately 2 mg of KI per 10 lb taken on the first day of radioiodine exposure and a protracted daily dose of approximately 1 mg KI per 10 lb taken based on the risk of continued exposure, to suppress ionizing radiation due to ^131^I [5]. A major caveat to this recommendation is that a KI prophylaxis regimen based on weight has not been investigated in a clinical setting. To reiterate, considering that a mg/pound recommendation may be too complicated for routine use we have built a table based on 15 mg tablets. However, while research supports a recommendation for adults weighing 150 lbs and above [5], currently, there is no data about dosages for neonates and children. The recommendation for KI prophylactic dose in children has been based on the scored 65 mg tablets [12], and it may not be effective at lower levels. Apart from examples from therapeutic exposures, the only resources for information about dosages for neonates and children are the experiences in Poland after Chernobyl [11] and presently Fukushima that have demonstrated that prophylaxis with KI tablets is far better than none. Dosage and formulations that are easily titrated and can extend the shelf—life of stable iodide require further investigation. 

Iodine 131 has several different practical uses, including applications in leak detection, hydraulic fracturing, medicine diagnostics and therapeutics, and ^131^I is a major byproduct from the nuclear fission of Uranium 235 [1,92,95,99] providing opportunity for terror events. Nevada nuclear test sites, Chernobyl, Fukushima, and the Handford Reservation Nuclear Production Complex provide examples of large releases of ^131^I [22,130] and provide impetus for the concern over future such accidents. The extent to which ^131^I disperses will depend upon the wind speed and direction, wind turbulence from solar heating, humidity, temperature, and the method of radiation release [51]. Radioactive plume dispersion occurs worldwide, far exceeding 300 miles previously mentioned [131]. This should implicate radiological hazard at distances otherwise overlooked. 

Mental health has been implicated as the greatest issue regarding nuclear accidents. The psychological effects due to nuclear accidents cause increased anxiety, a heightened perception of risk, subclinical depression and demoralization for six years following the event that did not return to normal until ten years after the incident [22]. These psychological effects and resultant physical detriments can be reduced significantly by the development and implementation of strong emergency preparedness plans. Preparing for psychosomatic disorders and addressing the panic and mass hysteria resultant of nuclear accidents is paramount to effective emergency response [132]. Notably, following the Chernobyl accident, evacuation of contaminated regions led to neuropsychiatric co-morbidities, psychosomatic problems, as well as other issues: including monetary burden and significant social unrested to ed from additionalco-morbidities associated with d. .ws of kinetics paper. insufficient individ [133].

These important considerations are the fruit of our scientific review regarding emergency response to ^131^I exposures:

- ^131^I, a primary byproduct of uranium-235 fission, is released from nuclear accidents and can be released in the event of a dirty bomb.
- Radiological plumes containing ^131^I cause benign and malignant thyroid nodules to develop within a 300 mile radius [49]; radiological plumes travel at an average speed of 10 mph.
- Two billion people are at risk for IDD the world’s leading cause of preventable brain damage [120]. Iodine deficiency disorder causes learning disabilities, cretinism and psychomotor impairment and can also result in IQ decreases of 10 to 15 points [119,120,121,122]. Importantly, iodine deficiencies predispose people to harmful effects of ^131^I exposure [17,21].
- Potassium iodide blocks the uptake of ^131^I by the thyroid. Ideally KI should be taken within two hours of ^131^I exposure to be effective. Prophylaxis is most successful if administered at the time of or just prior to exposure: 98%–99% effective. 1–2 h following exposure, prophylaxis with KI can be up to 85%–90% effective in blocking radioiodine.
- Potassium iodide prophylactic dosages should be based on the principles of a lowest effective prophylactic; dependent on a 15 mg, 7.5 mg scored, KI tablet; the rule of thumb is 1–2 mg KI per 10 lbs in weight. For euthyroid 150 lb adults 10 mg of stable iodine taken at radioiodine exposure is 87%–88% effective in blocking radioiodine and 30 mg of stable iodine is 98%–99% effective in blocking radioiodine [5].
- Seaweed, especially kelp, is the only alimentary alternative for prophylaxis; an associated limitation is the high variability in the iodine content of kelp. Studies of the pharmacokinetics of kelp could be investigated to accurately assess kelp as an alternative in prophylaxis. Interestingly, ^131^I uptake is reduced when the supply of alimentary iodine is increased [21,34,35,36].
- Pre-distribution of KI is necessary within 50 miles of any potential nuclear accident, and thyroid cancer risk areas extend 300 miles for children. This necessitates KI pre-distribution to all schools, hospitals and other of-interest sites extending 300 miles from any nuclear reactor. Evacuation or sequestering is impossible in congested urban areas. Evacuation protocols such as the plan recommended by the NYS Indian Point nuclear power plant do not extend past emergency planning zones and thus do not effectively address emergency procedures at distances exceeding 20 miles [132]. There is currently virtually no compliance with Senator Markey’s 20 miles radius KI pre-distribution law, section 127 of the Bioterrorism Act of 2002. In fact, there is little compliance with the 10 miles Ki pre-distribution radius law in the United States. Current dosage regimens are contributing to the failures to comply.
- Children, pregnant or lactating women, and people with iodine deficiencies are most vulnerable groups to the effects of radioiodine exposure. The best preparation for radiological emergencies is maintaining iodine sufficiency. By improving national and global iodine sufficiency, thyroid health as measured in thyroid nodule prevalence may be improved, and people will effectively mitigate vulnerability to radiation exposures. Iodine deficiency has been implicated as increasing the risk for the development of certain cancers: including breast, endometrial, ovarian, prostate, stomach, thyroid and uterus cancer [134,135,136]. Iodine sufficiency beyond adequacy would have the additional benefit of mitigating risk for the development of certain cancers, especially breast cancer. Iodine adequacy beyond sufficiency may provide protection against various diseases (*i.e.*, Iodine prophylaxis at higher iodine intake levels protects against radiation exposures). This same effect also occurs with other nutrients; for example, niacin sufficiency protects against pellagra, but only high doses beyond sufficiency can adequately lower cholesterol [137]. Nutritionally, some patients have iodide sufficiency, some are insufficient, and ultimately, iodide adequacy is measured by specific disease and radiation side effect prevention.
- Fast food and pre-prepared food industries can utilize iodized salt to help mitigate iodine deficiencies in at-risk regions. A country that is well iodized has additional thyroid reserves against thyroid radiation. An average euthyroid person contains 15–20 mg of iodine in their bodies, with ~75% aggregating in their thyroids [138]; the maximum iodine a thyroid can hold is upwards of 50 mg of iodine [139]. Higher thyroidal iodine content provides protection against radioiodine exposure.
- Following the Chernobyl nuclear accident, Poland utilizes KI in prophylaxis, reducing the estimated thyroid exposure by 40% in those distributed KI within 24 h [6]. Japan did not utilize KI for prophylaxis of the general public, acknowledging it was not prepared to act accordingly [66]. In the Polish radiological response to Chernobyl, a bolus dose of stable iodine was distributed to the 11 most effected regions; 15 mg KI was administered to newborns, 50 mg KI was given to children under 5 years of age, and 70 mg KI was distributed to all other age groups [11]. Without considering digestive adsorption issues; individual metabolic rates via the P450 drug metabolizing system; epigenetic effects; DNA polymorphic traits and other conditions, the above bolus dosage extrapolated for a week could result in as little as 2 mg KI/day for newborns, 7 mg KI/day for children 5 and under, and 10 mg KI/day for all other age groups. Single doses were advised by the authorities and multiple doses were not recommended [11]. Remarkably, this recommendation was effective [11]. A low dose, prolonged daily prophylaxis is well established and effective. Maintenance with lower dosages of KI is more effective in long-term thyroid dose aversion than large single doses [17]. In consideration with this recommendation, a 15 mg KI tablet would work for newborns. Approximately 6% of the Polish prophylaxis occurred prior to the decision to distribute KI to the public, through panic-driven, self-administering of iodine tincture [11]. Such occurrences necessitate improved education. It is very unlikely in a sophisticated U.S. population that individuals will trust a single dose to be effective for a prolonged period.
- There are 23 nuclear reactors with the same design as the Fukushima Dai-ichi nuclear power plant operating in the USA, 100 total operating nuclear power plants in the United States and 428 globally [9]. There is an inevitable and inherent risk that an accident to occur.
- Dosages as low as 20 mg KI, when administered at the time of radioiodine exposure, are 90% effective in blocking radioiodine intake [17]. Dosages of 1.8 mg KI in ~50 lb euthyroid children was up to 48% effective in thyroidal blockage, and dosages of 4.2 mg KI in euthyroid young adults was up to 69% effective in blocking radioiodine uptake [17]. Additional research should be conducted in infants, children and adolescents.
- The instructions of current KI prophylactic labels are unclear and may lead to over-dosing. Pro-KI, a 65 mg KI supplement for radiation protection, states that its protective effect lasts only ~24 h [140]. In instances of prolonged exposure risk, individuals will continue to take the high dosage KI supplements, which will increase the side effects from prophylaxis while not significantly increasing efficacy in thyroid blockage. Labels need to be made clear and accurate.

The ATA and WHO both endorse KI usage in a radiological event [9,13]. The ATA and responsible public agencies are encouraged to consider this list of considerations and these recommendations in their formulation and timely implementation of effective emergency response plans. Further research into these areas of concern, contingencies and requirements is urgently encouraged. Through personal communication with the NYS Commissioner of Homeland Secuirty and Emergency Service, Jerome Hauer, the distribution of KI is part of a broader preparedness plan (Figure A1).

## Figures and Tables

**Figure 1:**
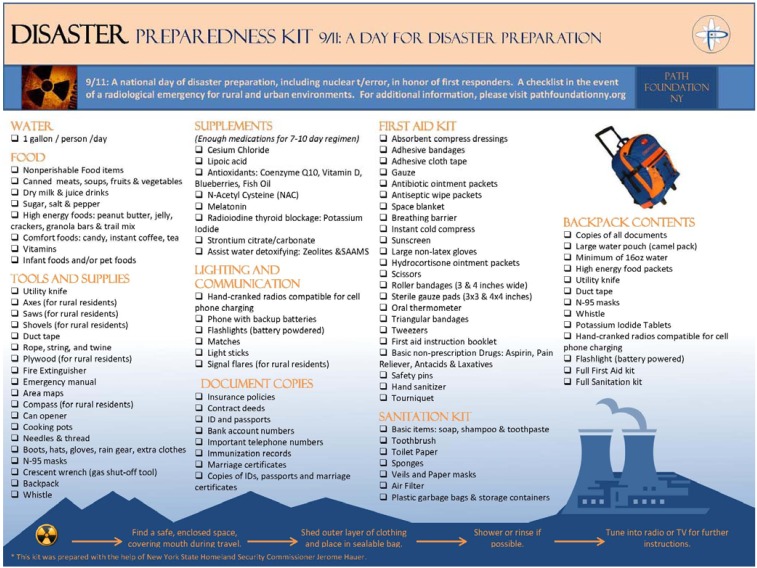
Disaster preparedness kit.

## Appendix

**Table A1:** Risk groups for iodine-induced hypothyroidism.

No underlying thyroid disease	Underlying thyroid disease	Other
*Fetus and neonate, mostly preterm*: Secondary to transplacental passage of iodine or exposure of neonate to topical or parenteral iodine-rich substances; *Infant*: Occasionally reported in infants drinking iodine-rich water (China); *Adult*: In Japanese subjects with high iodine intake where Hashimoto’s thyroiditis has been excluded; *Elderly*: Reported in elderly subjects with and without possible defective organification and autoimmune thyroiditis; *Chronic nonthyroidal illness*: Cystic fibrosis; Chronic lung disease (including Hashimoto’s thyroiditis); Chronic dialysis treatment; Thalassemia major; Anorexia nervosa.	*All ages*: Hashimoto’s thyroiditis; Euthyroid patients previously treated for Graves disease with ^131^I, thyroidectomy, or antithyroid drugs; Subclinical hypothyroidism; After transient postpartum thyroiditis; After subacute painful thyroiditis; After hemithyroidectomy for benign nodules. *Elderly*: subclinical hypothyroidism.	*All ages:* Euthyroid patients with a previous episode of amiodarone-induced destructive thyrotoxicosis; Euthyroid patients with a previous episode of interferon-alpha-induced thyroid disorders; Patients receiving lithium therapy.

*Source*: Modified from Risher, J.F.; Keith, L.S. *Iodine and Inorganiz Iodides: Human Health Aspects*; World Health Organization: Geneva, Switzerland, 2009. Available online: http://www.inchem.org/documents/cicads/cicads/cicad72.pdf. (accessed on 2 April 2014).

**Table A2:** Risk groups for iodine-induced hyperthyroidism.

Life Stage	Underlying thyroid disease	No underlying thyroid disease
All ages	Iodine supplementation for endemic iodine-deficiency goiter; Iodine administration to patients with euthyroid Graves disease, especially those in remission after antithyroid drug therapy; Nontoxic nodular goiter; Autonomous nodules; Nontoxic diffuse goiter.	Iodine administration to patients with no recognized underlying thyroid disease, especially in areas of mild to moderate iodine

*Source*: Modified from Risher, J.F.; Keith, L.S. *Iodine and Inorganiz Iodides: Human Health Aspects*; World Health Organization: Geneva, Switzerland, 2009. Available online: http://www.inchem.org/documents/cicads/cicads/cicad72.pdf. (accessed on 2 April 2014).

**Table A3:** Antidoting the confusion around radiation research.

**The curie (Ci) is replaced by the becquerel (Bq)**		**Becquerel (Bq) replaces the curie (Ci)**	
**Unit**	**Unit Conversion**	**Unit**	**Unit Conversion**
1 kilocurie (kCi) =	37 terabecquerel (TBq)	1 terabecquerel (TBq) =	~ 27 curie (Ci)
1 curie (Ci) =	37 gigabecquerel (GBq)	1 gigabecquerel (GBq) =	~ 27 millicurie (mCi)
1 millicurie (mCi) =	37 megabecquerel (MBq)	1 megabecquerel (MBq) =	~ 27 microcurie (µCi)
1 microcurie (µCi) =	37 kilobecquerel (kBq)	1 kilobecquerel (kBq) =	~ 27 nanocurie (nCi)
1 nanocurie (nCi) =	37 becquerel (Bq)	1 becquerel (Bq) =	~ 27 picocurie (pCi)
1 picocurie (pCi) =	37 millibecquerel (mBq)	1 becquerel (Bq) =	1s^-1^
**The rad (rad) is replaced by the gray (Gy)**		**The gray (Gy) replaces the rad (rad)**	
**Unit**	**Unit Conversion**	**Unit**	**Unit Conversion**
1 kilorad (krad) =	10 gray (Gy)	1 gray (Gy) =	100 rad (rad)
1 rad (rad) =	10 milligray (mGy)	1 milligray (mGy) =	100 millirad (mrad)
1 millirad (mrad) =	10 microgray (µGy)	1 microgray (µGy) =	100 microrad (µrad)
1 microrad (µrad) =	10 nanogray (nGy)	1 nanogray (nGy) =	100 nanorad (nrad)
**The roentgen (R) is replaced by coulomb/kg (C/kg)**		**Coulomb/kg (C/kg) replaces the roentgen (R)**	
**Unit**	**Unit Conversion**	**Unit**	**Unit Conversion**
1 kiloroentgen (kR) =	~ 258 millicoulomb/kg (mC/kg)	1 coulomb/kg (C/kg) =	~ 3,876 roentgen (R)
1 roentgen (R) =	~ 258 microcoulomb/kg (µC/kg)	1 millicoulomb/kg (mC/kg) =	~ 3,876 milliroentgen (mR)
1 milliroentgen (mR) =	~ 258 nanocoulomb/kg (nC/kg)	1 microcoulomb/kg (µC/kg) =	~ 3,876 microroentgen (µR)
1 microroentgen (µR) =	~ 258 picocoulomb/kg (pC/kg)	1 nanocoulomb/kg (nC/kg) =	~ 3,876 nanoroentgen (nR)
**The rem (rem) is replaced by the sievert (Sv)**		**The sievert (Sv) replaces the rem (rem)**	
**Unit**	**Unit Conversion**	**Unit**	**Unit Conversion**
1 kilorem (krem) =	10 sievert (Sv)	1 sievert (Sv) =	100 rem (rem)
1 rem (rem) =	10 millisievert (mSv)	1 millisievert (mSv) =	100 millirem (mrem)
1 millirem (mrem) =	10 microsievert (µSv)	1 microsievert (µSv) =	100 microrem (µrem)
1 microrem (µrem) =	10 nanosievert (nSv)	1 nanosievert (nSv) =	100 nanorem (nrem)

*Source*: Modified from Health Canada. Conversion Table. Environmental and Workplace Health. Available online: http://www.hc-sc.gc.ca/ewh-semt/occup-travail/radiation/dosim/res-centre/conversion-eng.php. (accessed on 9 November 2011).

**Table A4:** Radiation measurement units defined.

Radiation Measurement Unit	Definition	Measurement of Absorption or Emission
**Rem (rem)**	Measures biological effects of radiation absorbed by a non-radioactive substance	Absorption
**Sievert (Sv)**	Measures biological effects of radiation absorbed by a non-radioactive substance	Absorption
**Gray (Gy)**	Measures dose of radiation absorbed by a non-radioactive substance	Absorption
**Rad (rad)**	Measures dose of radiation absorbed by a non-radioactive substance	Absorption
**Becquerel (Bq)**	The level of radioactivity in a radioactive substance (radiation emitted)	Emission

*Source*: Modified from Health Canada. Conversion Table. Environmental and Workplace Health. Available online: http://www.hc-sc.gc.ca/ewh-semt/occup-travail/radiation/dosim/res-centre/conversion-eng.php. (accessed on 9 November 2011).

**Table A5:** Iodine status of pregnant and lactating women based on median urinary iodine.

Life Stage	Median urinary iodine (µg/L)	Status of Iodine Intake
Pregnant	≥500	Excessive
Pregnant	250–499	Above normal
Pregnant	150–249	Adequate
Pregnant	<150	Insufficient
Lactating	<100	Insufficient
Lactating	≥100	Adequate

*Source*: Modified from WHO (2011). Urinary Iodine Concentrations for Determining Iodine Status in Populations. Available online: http://apps.who.int/iris/bitstream/10665/85972/1/WHO_NMH_NHD_EPG_ 13.1_eng.pdf. (accessed on 31 March 2014).

**Table A6:** Iodine status of children 6–17 years of age based on median urinary iodine concentrations.

Life Stage	Median urinary iodine (µg/L)	Status of Iodine Intake
Child (6–17 years old)	<20	Severely insufficient
Child (6–17 years old)	20–49	Moderately insufficient
Child (6–17 years old)	50–99	Mildly insufficient
Child (6–17 years old)	100–199	Adequate
Child (6–17 years old)	200–299	Above normal
Child (6–17 years old)	≥300	Excessive

*Source*: Modified from WHO (2011). Urinary Iodine Concentrations for Determining Iodine Status in Populations. Available online: http://apps.who.int/iris/bitstream/10665/85972/1/WHO_NMH_NHD_EPG_ 13.1_eng.pdf. (accessed on 31 March 2014).
