# Correction: Braverman, E.R., *et al.* Managing Terrorism or Accidental Nuclear Errors, Preparing for Iodine-131 Emergencies: A Comprehensive Review. *Int. J. Environ. Res. Public Health* 2014*,* *11,* 4158–4200

**DOI:** 10.3390/ijerph110807803

**Published:** 2014-08-04

**Authors:** Eric R. Braverman, Kenneth Blum, Bernard Loeffke, Robert Baker, Florian Kreuk, Samantha Peiling Yang, James R. Hurley

**Affiliations:** 1 Department of Psychiatry, College of Medicine, University of Florida and McKnight Brain Institute, Gainesville, FL 32610, USA; E-Mail: ericb1957@gmail.com; 2 Department of Clinical Neurology, PATH Foundation NY, New York, NY 10010, USA; E-Mails: helpingotherstoday@comcast.net (B.L.); fkreuk.path@gmail.com (F.K.); 3 Department of Neurosurgery, Weill Cornell Medical College, New York, NY 10065, USA; 4 Department of Biological Sciences, Texas Tech University, Lubbock, TX 79409, USA; E-Mail: Robert.Baker@ttu.edu; 5 Natural Science Research Laboratory, Museum of Texas Tech University, Lubbock, TX 79409, USA; 6 Department of Endocrinology, National University Hospital of Singapore, Singapore 119228; E-Mail: Peiling_Yang@nuhs.edu.sg; 7 Department of Medicine, Weill Cornell Medical College, New York, NY 10065, USA; E-Mail: jrh2004@med.cornell.edu

The authors wish to make the following amendments to their paper published in *International Journal of Environmnetal Research and Public Health* [1]:

1. Page 4170, line 11, the sentence “The continental wide average wind speed at altitude of 80 miles falls between 8 and 23.5 miles/h [54]. Therefore, in a 24 h period radioactive plumes can disperse anywhere in the continental United States between 192 to 564 m, assuming…” should read “The continental wide average wind speed at an altitude of 80 meters falls between 8 and 23.5 miles/h [54]. Therefore, in a 24 h period radioactive plumes can disperse anywhere in the continental United States between 192 to 564 miles, assuming...”.
2. Page 4176, Table 5, the items under “Governmental Body/Agency”: “Federal Emergenct Management Agency” should read “Federal Emergency Management Agency”, “Food and Drug Agency” should read “Food and Drug Administration”. The correct Table 5 should be:

**Table 5:** Governmental body/agency, KI usage and emergency preparedness recommendations.

Governmental Body/Agency	Distribution Recommended	Pre-Distribution Recommended	Pre-Distribution Effectiveness	Pre-Distribution Distance	KI Dose
United States of America	Yes	Yes	N/A	20 miles	FDA-Endorse
New Jersey State	Yes	Yes at public education & Distribution sessions	~10%	10 miles EPZ	FDA-Endorse
New York State	Yes	Distribution by county; Pick-up locations; Via mail	15% in EPZ	Offered KI regardless of distance	FDA-Endorse
World Health Organization	Yes	Yes	N/A	N/A	N/A
United States Nuclear Regulatory Commission	Yes	Yes	N/A	10 miles radius	FDA-Endorse
American Thyroid Association	Yes	Yes	N/A	50 miles Pre-Distribution; 50–200 miles Stockpile Local Public Facilities; >200 miles National stockpile	FDA-Endorse
Food and Drug Administration	Yes	Yes	N/A	10 miles radius of USNRC mentioned	
Centers for Disease Control and Prevention	N/A	N/A	N/A	Public health or Emergency managers to decide	FDA-Endorse
Federal Emergency Management Agency	Yes	Yes	N/A	10 miles radius	FDA-Endorse

Notes: EPZ—Emergency Planning Zone; N/A—Not Applicable; FDA-Endorse—The governmental body/agency endorses the FDA’s recommendations for KI dosage.

The authors would like to apologize for any inconvenience caused to readers by these changes.
